# Supplementary material for: Mechanosensory neurons control sweet sensing in Drosophila
Source: Nat Commun. 2016 Sep 19;7:12872. doi: 10.1038/ncomms12872 (PMC5031804; doi:10.1038/ncomms12872)
Supplement: Supplementary Information — Supplementary Figure 1-4 and Supplementary Table 1. [file ncomms12872-s1.pdf]

Supplementary Figure 1

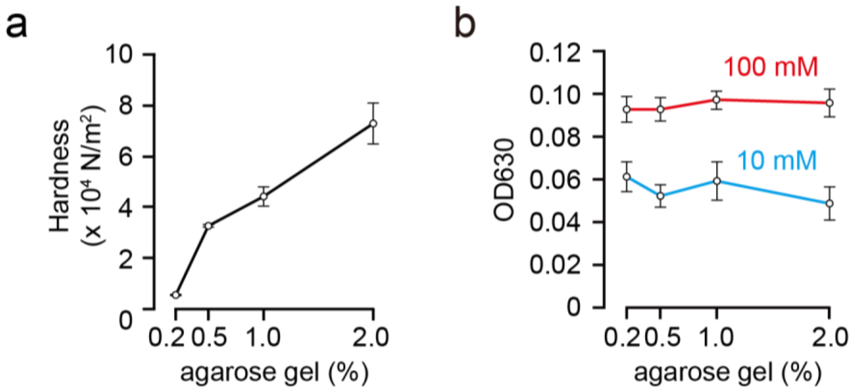

Supplementary Figure 1. Food hardness and food palatability. (**a**) Food hardness depending on agarose concentration. n=6. (**b**) Mean food intake of 10 mM and 100 mM sucrose-containing food depending on its hardness. n=6. All data are presented as means ± S.E.M. The ANOVA was used to determine statistical significance.

Supplementary Figure 2

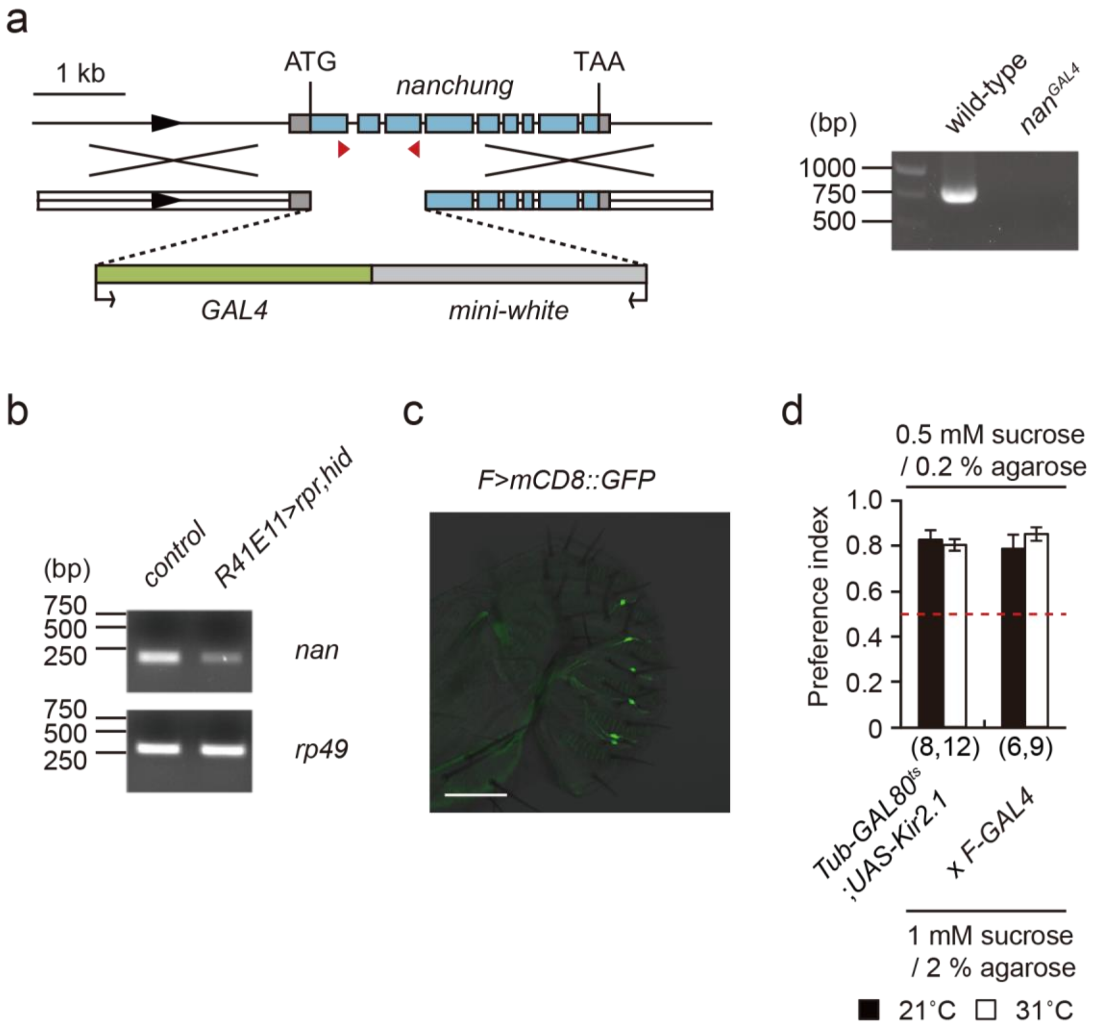

Supplementary Figure 2. Generation of the *nan*^{GAL4} allele and labellar mechanosensory neuron *nan* expression. (**a**) Structure of the *nan* locus and homologous recombination targeting scheme used to generate the *nan*^{GAL4} allele (left). Blue boxes indicate exons. Red triangles indicate the primers used for genomic DNA PCR. Deletion of *nan* confirmed by genomic DNA PCR (right). **(b)** Expression of *nan* in the labellum as measured by RT-PCR on labella from control and mechanosensory neuron-ablated flies. *rp49* was used as an internal control. (**c**) Confocal images of labella expressing mCD8::GFP driven by *F-GAL4*. Labella were stained with a rabbit anti-GFP. GFP fluorescence was superimposed on a transmitted light image. Scale bar represents 50 μm. **(d)** Food preference of *F-GAL4* neuron-inactivated

flies. Flies were given a choice between 0.5 mM sucrose in 0.2% agarose and 1 mM sucrose in 2 % agarose. Flies were raised at 21˚C and shifted to the indicated temperature for 3 days before assaying. n is indicated in parentheses. The unpaired *t*-test was used to determine statistical significance. All data are presented as means ± S.E.M.

Supplementary Figure 3

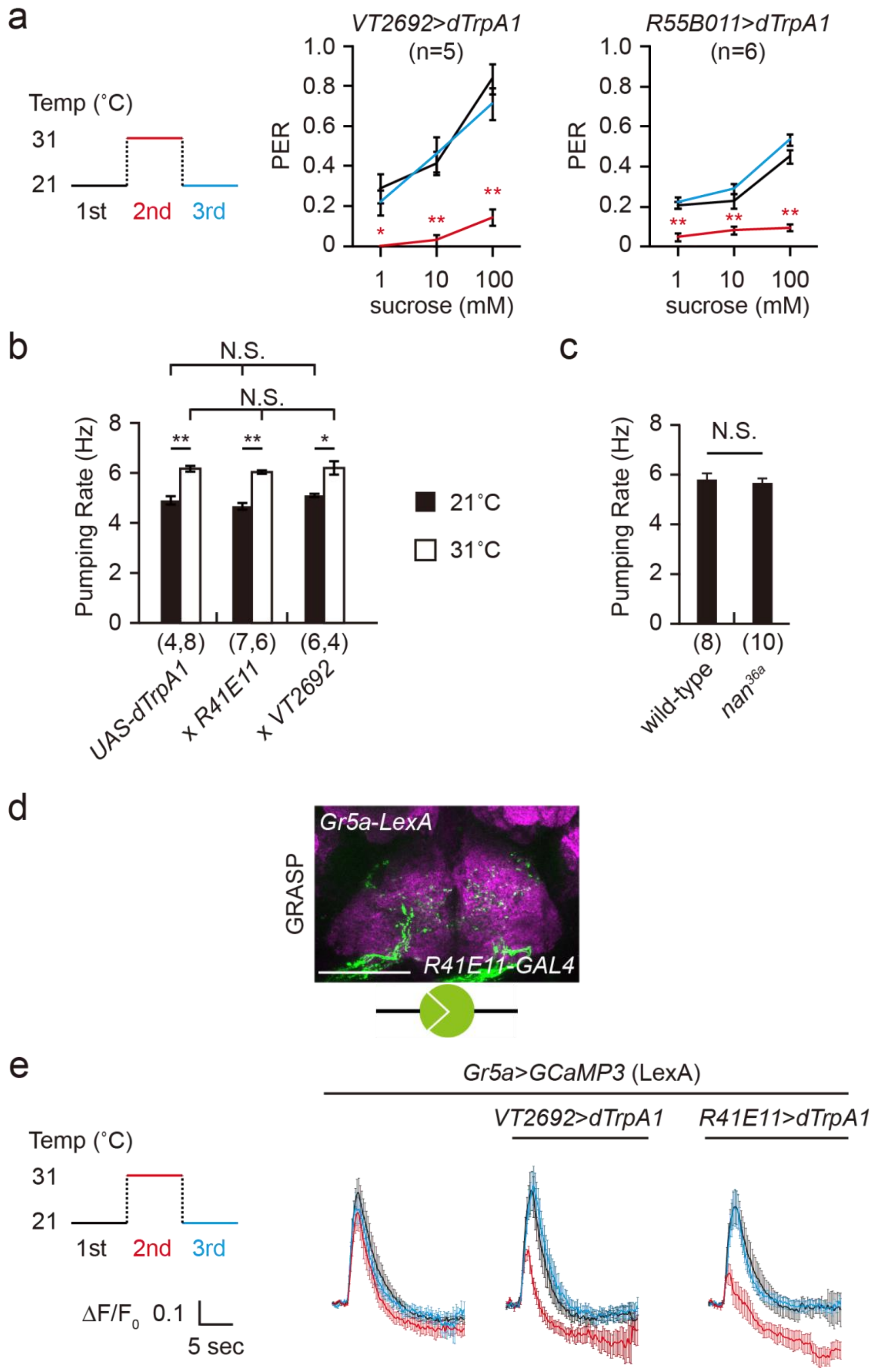

Supplementary Figure 3. Functional interaction between mechanosensory neurons and sweet GRNs in the SEZ. (**a**) Proboscis extension response (PER) upon activation of mechanosensory neurons. The PER assay was performed at the indicated temperatures. ANOVAs with Tukey *post-hoc* tests. $^{*}P < 0.05$, $^{**}P < 0.01$. (**b,c**) Effects of mechanosensory neurons and Nan on pharyngeal pumping. (**b**) Pumping frequencies upon activation of mechanosensory neurons. Black and white bars indicate the pumping frequencies (Hz) of the indicated genotypes at 21˚C and 31˚C, respectively. The unpaired *t*-test was used to determine the statistical significance of changes in frequency related to temperature. $^{*}P < 0.05$, $^{**}P < 0.01$. The ANOVA revealed no significant changes with respect to genotype. N.S. means no significance. (**c**) Pumping frequencies of *wild-type* and nan^{36a}. The unpaired *t*-test was used to determine statistical significance. (**d**) Structural proximity of mechanosensory neurons and sweet GRNs in the subesophageal zone (SEZ). GRASP images in the SEZ (above) and GRASP schematics (below). GRASP between mechanosensory neurons labeled with *R41E11-GAL4* and sweet GRNs labeled with *Gr5a-LexA*. The genotype of the GRASP flies was *Gr5a-LexA/+;LexAOp-CD4::spGFP*$_{11}$/+;*UAS-CD4::spGFP*$_{1\text{-}10}$/*R41E11-GAL4*. nc82 neuropil staining (magenta). Scale bar represents 50 μm. (**e**) Mechanosensory neurons inhibit calcium responses in the axon termini of sweet GRNs. Time course of calcium responses in the axon termini of sweet GRNs in the SEZ shown as average $\Delta F/F_0$ traces ± S.E.M. The 1st and 3rd trials were conducted at 21˚C and 2nd trial was conducted at 31˚C to activate mechanosensory neurons via dTrpA1. All data are presented as means ± S.E.M.

Supplementary Figure 4

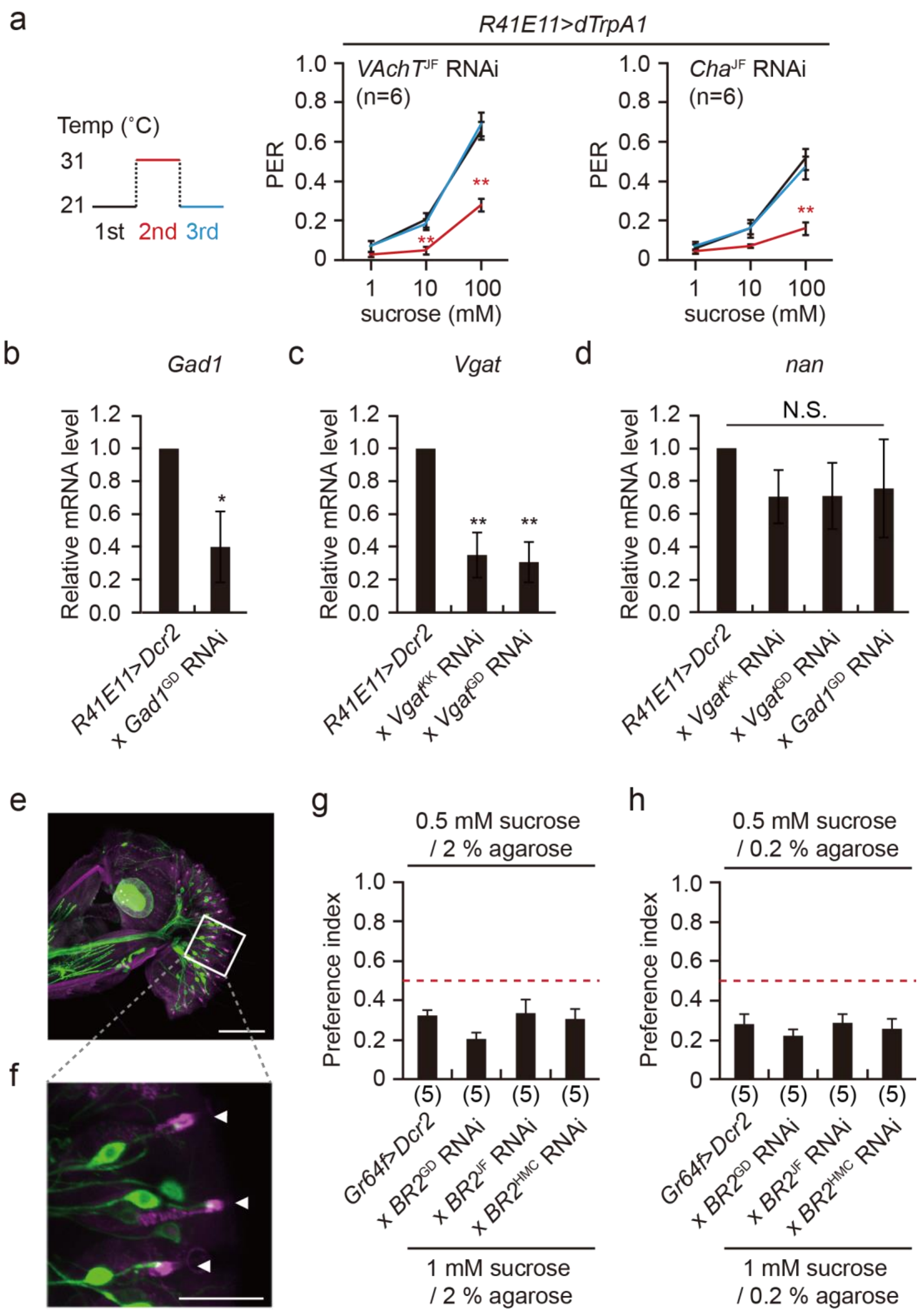

Supplementary Figure 4. Labellar mechanosensory neurons are GABAergic. (**a**) PER of flies upon knockdown of cholinergic components in mechanosensory neurons. The PER assay was conducted at the indicated temperatures. n=6. ANOVAs with Tukey *post-hoc* tests. (**b-d**) Knockdown efficiency and specificity of *Gad1* and *Vgat* RNAi in labellar mechanosensory neurons. Relative expression of *Gad1* (b), *Vgat* (c), and *nan* (d) transcripts upon knockdown of *Gad1* and *Vgat*. Each C_T value is normalized to the C_T value for the *rp49* internal control. n=3 biological replicates with technical duplicates. Unpaired Student's *t*-tests (b) or ANOVAs with Tukey *post-hoc* tests (c,d). (**e,f**) Representative confocal images of *Vgat-GAL4*>mCD8::GFP labella double-stained with a mouse GFP antibody (green) and a rabbit NOMPA antibody (magenta) to visualize the sensillar base. (**f**) High magnification view of the white boxed area in (e). White arrowheads indicate sensilla base marked with NOMPA antibody. Scale bar represents 50 μm. (**g,h**) Food preference between 0.5 mM and 1 mM sucrose either in (g) 2% agarose or (h) 0.2% agarose. The red dashed line at 0.5 represents no preference for either of the two food sources. n=5. ANOVA., and All the data are presented as means ± S.E.M. $^{*}P < 0.05$, $^{**}P < 0.01$, and N.S. indicates not statistical significance.

Supplementary Table 1

	Number of mechanosensory neurons	Number of GFP positive neurons		
		R41E11 (n=13)	*VT2692* (n=8)	*R55B01* (n=14)
labellum	63	58.85±1.02	60.86±1.14	26.07±3.10
taste bristle	31	27.62±1.03	29.13±1.29	22.21±2.47
lateral mechanosensilla	2	2±0.00	2±0.00	2±0.00
taste peg	30	29.23±0.97	29.75±1.01	1.86±0.70

Supplementary Table 1. Cells labeled by various mechanosensory *GAL4* drivers Average GFP positive neurons labeled by each mechanosensory neuron driver per half of the labellum. *UAS*-mCD8::*GFP* was used as a reporter. Total mechanosensory cells per taste bristle, labellar mechanosensillum, and taste peg are also indicated. Data are means ± S.E.M.
